# Fecal Microbiome Changes and Specific Anti-Bacterial Response in Patients with IBD during Anti-TNF Therapy

**DOI:** 10.3390/cells10113188

**Published:** 2021-11-16

**Authors:** Dagmar Schierova, Radka Roubalova, Martin Kolar, Zuzana Stehlikova, Filip Rob, Zuzana Jackova, Stepan Coufal, Tomas Thon, Martin Mihula, Martin Modrak, Miloslav Kverka, Lukas Bajer, Klara Kostovcikova, Pavel Drastich, Jana Hercogova, Michaela Novakova, Martin Vasatko, Milan Lukas, Helena Tlaskalova-Hogenova, Zuzana Jiraskova Zakostelska

**Affiliations:** 1 Institute of Microbiology of the Czech Academy of Sciences, 142 20 Prague, Czech Republic; dagmar.schierova@biomed.cas.cz (D.S.); r.roubalova@biomed.cas.cz (R.R.); zuzana.stehlikova@biomed.cas.cz (Z.S.); zuzana.jackova@biomed.cas.cz (Z.J.); coufal@biomed.cas.cz (S.C.); tomas.thon@biomed.cas.cz (T.T.); martin.mihula@biomed.cas.cz (M.M.); martin.modrak@biomed.cas.cz (M.M.); kverka@biomed.cas.cz (M.K.); bajl@ikem.cz (L.B.); klimesov@biomed.cas.cz (K.K.); tlaskalo@biomed.cas.cz (H.T.-H.); 2 IBD Clinical and Research Centre ISCARE a.s., 190 00 Prague, Czech Republic; martin.kolar@gmail.com (M.K.); vasatkomartin@seznam.cz (M.V.); milan.lukas@email.cz (M.L.); 3 Dermatovenerology Department, Second Faculty of Medicine, University Hospital Bulovka, Charles University in Prague, 180 81 Prague, Czech Republic; filip.rob@bulovka.cz (F.R.); jana.hercogova@gmail.com (J.H.); michaela.novakova@bulovka.cz (M.N.); 4 Institute for Clinical and Experimental Medicine of the Czech Academy of Science, 140 21 Prague, Czech Republic; drastich@hotmail.com; 5 Institute of Medical Biochemistry and Laboratory Diagnostics, General University Hospital and First Faculty of Medicine, Charles University in Prague, 128 08 Prague, Czech Republic

**Keywords:** inflammatory bowel disease, biological therapy, tumor necrosis factor-α, microbiome, mycobiome

## Abstract

Inflammatory bowel diseases (IBD) are chronic disorders of the gastrointestinal tract that have been linked to microbiome dysbiosis and immune system dysregulation. We investigated the longitudinal effect of anti-TNF therapy on gut microbiota composition and specific immune response to commensals in IBD patients. The study included 52 patients tracked over 38 weeks of therapy and 37 healthy controls (HC). To characterize the diversity and composition of the gut microbiota, we used amplicon sequencing of the V3V4 region of 16S rRNA for the bacterial community and of the ITS1 region for the fungal community. We measured total antibody levels as well as specific antibodies against assorted gut commensals by ELISA. We found diversity differences between HC, Crohn’s disease, and ulcerative colitis patients. The bacterial community of patients with IBD was more similar to HC at the study endpoint, suggesting a beneficial shift in the microbiome in response to treatment. We identified factors such as disease severity, localization, and surgical intervention that significantly contribute to the observed changes in the gut bacteriome. Furthermore, we revealed increased IgM levels against specific gut commensals after anti-TNF treatment. In summary, this study, with its longitudinal design, brings insights into the course of anti-TNF therapy in patients with IBD and correlates the bacterial diversity with disease severity in patients with ulcerative colitis (UC).

## 1. Introduction

Inflammatory bowel diseases (IBD) such as Crohn’s disease (CD) and ulcerative colitis (UC) are chronic disorders with an unknown etiology and mechanism of progression. However, several associated risk factors have been identified and extensively studied, ranging from genetic predisposition and environmental factors to immune system dysregulation [1]. Biological therapy is a standard part of the treatment regimen for IBD in the Czech Republic and worldwide. Specifically, two of the currently available monoclonal antibodies for IBD treatment include antibodies against tumor necrosis factor-α (TNF-α), infliximab (IFX), a chimeric monoclonal antibody, and adalimumab (ADA), a fully human monoclonal antibody IBD [2].

TNF-α is a proinflammatory cytokine, which is typically increased in patients with IBD [3,4]. It can induce gut epithelial cell damage by mediating apoptosis [5], affecting tight junction proteins [6], and promoting the secretion of other inflammatory cytokines, further exacerbating the pathological processes [7].

The human gut is populated by a vast number of microorganisms dominated by bacteria. Multiple bacterial phyla are commonly found in the human gut, such as Firmicutes, Bacteroides, Proteobacteria, Actinobacteria, and Verrucomicrobia, with the first two being the most prevalent. Changes in the relative abundance of taxa within these groups as well as in overall community diversity are commonly reported in studies on patients with IBD. Associations of healthy and diseased states with taxa on various taxonomical levels have been shown with somewhat conflicting results, but dysbiosis has been documented consistently in IBD individuals [8,9].

Since responsiveness to anti-TNF treatment varies among patients with IBD, there have been substantial efforts to identify microbial biomarkers, which could have predictive value. One of the early studies identified that lower abundance of *Faecalibacterium prausnitzii* and *Clostridium coccoides* could predict clinical relapse within one year after IFX treatment discontinuation [10]. A similar study found that *Escherichia coli* was significantly decreased in patients with CD after ADA treatment [11]. Both these pioneering studies used qPCR quantification limited to a handful of bacterial taxa. Another study tracked a panel of predefined bacteria in patients with UC following anti-TNF therapy, of which only *Faecalibacterium prausnitzii* was identified as a discriminatory marker between therapy responders and non-responders [12]. Studies using more robust methods such as amplicon sequencing followed. The presence of several genera, including *Bifidobacterium*, *Collinsella*, *Lachnospira*, *Roseburia*, *Eggerthella,* [13] and phylum *Lachnospiraceaae* [14] was associated with successful anti-TNF treatment outcomes. In addition, a co-occurrence analysis revealed a cluster of bacterial taxa, whose disruption is associated with disease severity, therapy failure, unhealthy lifestyle, and probability of relapse. It appears that the common denominator of bacteria in this cluster is short-chain fatty acid (SCFA) synthesis [13]. A different study also using amplicon sequencing showed that after thirty weeks of anti-TNF treatment, the gut microbiome composition in a group of patients with IBD shifted towards that of healthy controls. *Coproccocus* and *Roseburia*, both SCFA producers, were significantly reduced in patients with IBD at baseline [15]. A recent study revealed the order *Actinomycetales* to be differentially abundant in patients with IBD pre- and post-IFX treatment, regardless of the degree of their response to the therapy [16].

Fungi in the gastrointestinal tract are much more scarce compared to bacteria; it is estimated that less than 0.1% of gut microbial genes are of eukaryotic origin [17]. The most prevalent genera commonly found in the human gut include *Aspergillus, Candida, Debaryomyces, Malassezia, Penicillium,* and *Saccharomyces* [18,19,20,21]. Contrary to bacterial diversity, which is usually decreased in patients with IBD, fungal abundance and diversity tend to be increased [19,22,23], but one study also reported a reduced diversity [20].

Dysbiosis in patients with IBD is quite well documented across many studies [10,15,24,25]. However, studies linking fluctuations in diversity and specific microbial phylotypes with disease advancement after anti-TNF therapy do not provide consistent results. Therefore, the aim of this study was to track changes in the bacterial and fungal microbiome over the course of anti-TNF therapy and link it to disease severity. Moreover, we studied various immune factors related to the clinical status of patients with IBD that might contribute to the observed microbial diversity changes.

## 2. Materials and Methods

### 2.1. Patients and Sample Collection

Patients were recruited at the ISCARE Clinical and Research Centre for Inflammatory Bowel Disease in the Czech Republic from November 2018 to December 2020. This study included patients diagnosed with IBD according to the guidelines of the European Crohn’s and Colitis Organization (ECCO) [26], who had started to receive anti-TNF therapy. Fecal and blood (serum, peripheral blood mononuclear cell isolation) samples were collected before the beginning of therapy (baseline, week 0) and at each patient visit (weeks 2, 8, 14, 20, 26, 32, and 38) as described previously [27]. All samples were stored at −80 °C until processing. At each visit, we recorded medical history, disease severity, responsiveness to treatment, and various clinical parameters: Harvey–Bradshaw index (HBI) in patients with CD and partial Mayo score (pMayo) in patients with UC; C-reactive protein (CRP); fecal calprotectin (FC); ferritin; hemoglobin (Hb); platelet count (PLT); white blood cells (WBC). HBI and pMayo were calculated by a specialized gastroenterologist during patient visits at the outpatient clinic according to disease activity reflected by patient outcomes and physician’s assessment [28,29]. Healthy control subjects were recruited at the Institute of Clinical and Experimental Medicine (IKEM) in the Czech Republic and comprised 37 individuals. The exclusion criteria for healthy subjects were a gastrointestinal or dermatologic diagnosis and use of antibiotics within 3 months prior to sampling. All study participants signed informed consent forms. This study was approved by the Ethics Committees of ISCARE (Nr2015/Ia) and IKEM (Nr2015/Ia).

### 2.2. DNA Extraction, PCR Amplification, Sequencing, and Data Analysis

DNA was isolated from the stool specimen using the ZymoBIOMICS DNA Miniprep Kit (Zymo Research, Irvine, CA, USA) according to the manufacturer’s protocol. The V3V4 region of 16S rRNA gene and the fungal ITS1 region covered by specific primers with barcodes (341F GTCCTACGGGNGGCWGCAG and 806R GGACTACHVGGGTWTCTAAT) and (F GTAAAAGTCGTAACAAGGTTTC and R AAGTTCAAAGAYTCGATGATTCAC), respectively, were chosen as representative sequences for taxonomic identification. The amplification reaction was performed with the KAPA HiFi HotStart Ready Mix (Roche, Penzberg, Germany), as follows: initial denaturation step 3 min at 95 °C followed by 25 cycles at 95 °C for 30 s, 55 °C for 30 s, 72 °C for 30 s with a final elongation step at 72 °C for 5 min using 5 ng/µL DNA. PCR products were checked using QIAxcel advanced capillary electrophoresis (QIAgen, Hilden, Germany). Triplicates of the amplicons were pooled and normalized with the SequalPrep™ Normalization Plate Kit (ThermoFisher Scientific, Waltham, MA, USA), concentrated (Eppendorf centrifugal vacuum concentrator), and purified with DNA Clean & Concentrator Kit (Zymo Research, Irvine, CA, USA). Subsequently, the amplicon libraries were ligated with sequencing adapters using the KAPA HyperPlus Kit (Roche, Penzberg, Germany), pooled in equimolar concentrations, and sequenced. Amplicon sequencing was performed using the Miseq platform (Illumina, San Diego, CA, USA).

Reads were quality filtered with Cutadapt (version 1.15), joined with Fastq-join (version 1.3), and demultiplexed using a custom R script. The sequences were trimmed and denoised, and amplicon sequence variants (ASVs) were generated with the Qiime2 plugin DADA2. Qiime2 (version 2021.2) [30] pipeline was used to calculate alpha and beta diversities. Bacterial taxonomy was assigned with VSEARCH classifier against the SILVA database (release 138) with 99% similarity. Only forward reads were used with the ITS sequences and fungal taxonomy was assigned with blast against the Unite database (version 8) with 97% similarity. The MetaCyc metabolic pathways were predicted from the 16S-based taxonomic profiles with the aid of the Qiime2 plugin Phylogenetic Investigation of Communities by Reconstruction of Unobserved States (PICRUSt2) [31].

### 2.3. Preparation of Bacterial Lysates

The bacteria were obtained from DSMZ (Deutsche Sammlung von Mikroorganismen und Zellkulturen GmbH, Braunschweig, Germany) and cultured as recommended. Specifically, *Lactobacillus plantarum* CCDM 182 (ATCC Medium No. 416); *Bifidobacterium adolescentis* CCUG 18363 (DSMZ medium No. 58.); *Blautia coccoides* (ATCC Medium No. 1490); *Roseburia intestinalis* L1-82 (ATCC Medium No. 2695); *Eubacterium rectale* ATCC 3365 (ATCC Medium No 1703); *Faecalibacterium prausnitzii* A2-165 (DSMZ medium No. 1611); *Ruminococcus flavefaciens* (ATCC Medium No. 1365 E); *Bacteroides thetaiotaomicron* VPI 5482 (ATCC Medium No. 1490); *Prevotella ruminicola* M384 (ATCC Medium No 1703); *Escherichia coli* K6 (Merck, Kenilworth, NJ, USA, L3022). All bacteria except *Escherichia coli* K6 were grown under anaerobic conditions. Bacterial lysates were prepared as previously described and used for either PBMCs stimulation or for antigen coating in an indirect ELISA [32,33].

### 2.4. Indirect Enzyme-Linked Immunosorbent Assays (ELISAs)

Serum concentrations of total immunoglobulin G (IgG), immunoglobulin A (IgA), and immunoglobulin M (IgM) were determined using commercially available kits according to manufacturers’ instructions (Invitrogen, Waltham, MA, USA). The serum concentrations of anti-bacterial antibodies of IgM, IgG, and IgA isotypes were analyzed by an in-house developed indirect ELISA as described previously [27]. All serum samples were diluted at 1:200. Antibody levels are reported in arbitrary units (AU), where a reference serum sample was applied on each ELISA plate and its mean value of OD (450–650 nm) was used as thousand arbitrary unit (1000 AU).

### 2.5. Peripheral Blood Mononuclear Cells (PBMCs)

PBMCs were isolated and cultivated as previously described [27]. Interleukin-17A (IL-17A) in supernatants of PBMCs stimulated by bacterial antigens was quantified by a commercially available ELISA kit (R&D Systems, Minneapolis, MN, USA).

### 2.6. Statistical Analysis

Differences in alpha diversity were evaluated using non-parametric tests, specifically the Mann–Whitney-U test or Kruskal–Wallis test for multiple group comparison and Wilcoxon signed-rank test for paired comparisons. A Benjamini and Hochberg procedure was applied to control the false discovery rate. The relationship between alpha diversity and disease severity was assessed using the Spearman correlation. Beta diversity variance was determined by the qiime2 plugin ADONIS [30]. For beta diversity comparisons, permutation tests including PERMANOVA and PERMDISP (with 999 permutations) were used. Beta diversity similarities of HC and patients with IBD at baseline and at the endpoint of the study were assessed by a restricted permutational design implemented in the ADONIS function from the R package Vegan (version 4.0). Differential abundance analysis was performed with the aid of ANCOM v2.1, a modified version of the original ANCOM [34], which allows paired design and adjustment for covariates. For diversity analysis, reads were rarefied to the minimum sample depth. For taxonomic analysis, unrarefied sequences were used. Antibody and cytokine levels were log-transformed and compared with one-way ANOVA or Student’s *t*-test along with Tukey multiple comparison test. Graphs were generated using python 3.9. with the packages Matplotlib, Seaborn, and Sklego and in GraphPad Prism (version 9.1.2).

## 3. Results

### 3.1. Cohort Characteristics

In the microbiome study, 52 patients suffering from IBD (34 CD, 18 UC) and 37 healthy controls were observed longitudinally. A total of 352 samples from 89 individuals at several time points were collected. Out of the 52 patients who brought their fecal sample at baseline (week 0), only 27 (17 patients with CD and 10 patients with UC) reached the endpoint at week 38. The clinical data for patients who participated in the study until week 38 and healthy controls are summarized in Table 1. The mean age at diagnosis was very similar in patients with CD and UC. The sex ratio was skewed towards females in both HC and patients with IBD (Table 1). Basic clinical parameters, such as CRP, WBC, PLT, ferritin, Hb, and FC were measured (Table 1) and the elevated levels point to an ongoing inflammation in the patients with IBD. At baseline (week 0) 65% (22/34) of patients with CD were in clinical remission but no patient from the patient group with UC was in remission (0/18).

### 3.2. Individual and Baseline Differences in Gut Bacteriome of Healthy Controls and Patients with IBD

**Figure 1:**
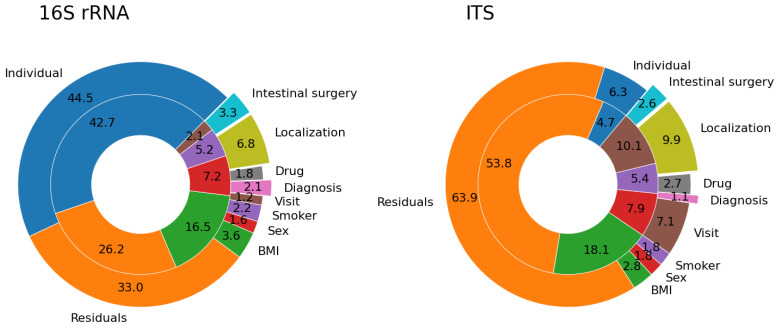
Proportion of beta diversity variance explained by our metadata categories. The pie charts show the percentages of variance in Bray-Curtis dissimilarity explained by different metadata categories in healthy controls (inner ring) and patients with IBD (outer ring). Data for both bacterial (16S rRNA) and fungal (ITS) microbiome are shown. Labels in the outer and inner rings share the same color code.

Healthy controls comprised 37 individuals, 12 of them subjected to repeated sampling. The bacterial alpha diversity represented by Shannon entropy was significantly different (*p* < 0.001) among healthy individuals (Figure S1A). The high interindividual variability was also evident in beta diversity dissimilarities represented by the Bray–Curtis metric (Figure 1). Forty-three percent of the overall variance can be attributed to the individual, whereas only 2% to the time of sampling (visit). The residual variance of 26% includes the variance of each individual in time, sequencing variance, or variance not explained by our metadata categories. Importantly, in the healthy controls group, there is no significant temporal variation in bacterial diversity (Figure S2).

Similarly to healthy controls, patients with IBD differed in both alpha (Figure S1B,C) and beta diversity. The individual factor explained almost half of the bacterial beta diversity variance (46%). The time factor (visit) explained only 1% of the variance, diagnosis 2%, localization of the disease 7%, and 33% of the observed variance could not be explained by our metadata categories (Figure 1).

**Figure 2:**
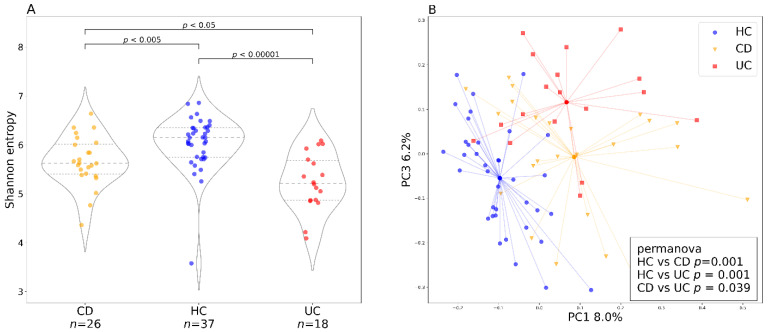
Baseline bacterial alpha and beta diversity for the two IBD groups and healthy controls. (**A**) baseline (week 0) bacterial alpha diversity represented by Shannon entropy plotted for Crohn’s disease and ulcerative colitis as well as healthy controls. In the violin plots, the middle dashed line represents the median and the outer dashed lines represent the first and third quartiles. (**B**) PCoA plot shows the bacterial beta diversity represented by the Bray–Curtis metric for the three groups (Crohn’s disease, ulcerative colitis, and healthy controls) at baseline (week 0). The dots represent samples at baseline and the lines are connecting individual samples with cluster centroids. The box in the lower right corner shows the results of the PERMANOVA test with 999 permutations. Abbreviations: CD, Crohn’s disease; HC, healthy controls; UC, ulcerative colitis; *n*, number of samples.

The bacterial diversity at baseline (week 0) differed between patients with CD and UC and healthy controls (Figure 2). Bacterial alpha diversity represented by Shannon entropy was highest in the HC group and lowest in the UC group (Figure 2A). Bacterial beta diversity represented by Bray–Curtis distance is illustrated by PCoA (Figure 2B). Statistically significant differences between the cluster centroids are supported by PERMANOVA and PERMDISP tests, but the contribution of diagnosis to the overall beta diversity variation is quite small.

### 3.3. Factors Influencing Bacterial Diversity

**Figure 3:**
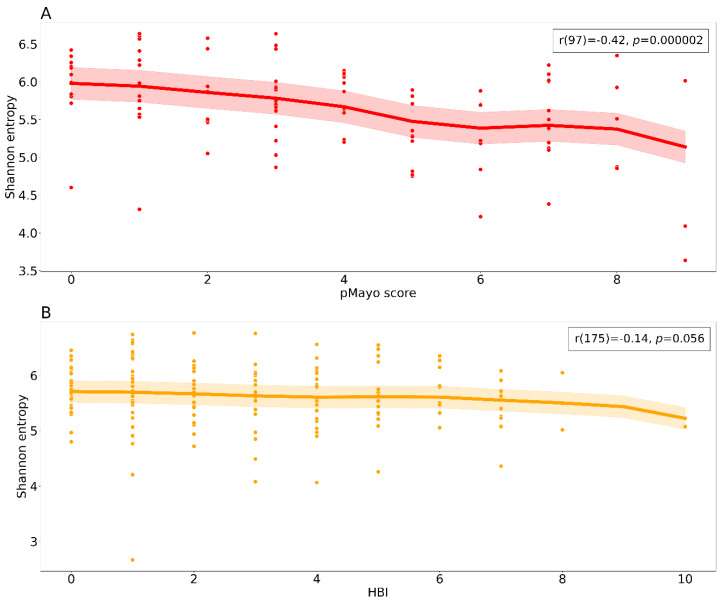
Relationship between alpha diversity and clinical scores. The scatter plots show a negative correlation between the clinical scores and Shannon entropy for both diagnostic groups (**A**) ulcerative colitis and (**B**) Crohn’s disease. Each dot represents a sample from a patient at a specific time point including the baseline. The bold lines represent locally weighted regression with a confidence interval fill. Spearman correlation results are reported in the upper right box. Abbreviations: pMayo, partial Mayo score; HBI, Harvey–Bradshaw index.

Both patients with CD and UC experienced changes in bacterial alpha diversity and disease severity during the study. These changes were more pronounced in the patient group with UC (Figure S3). In patients with UC, we observed a negative association between the clinical score and Shannon entropy with bacterial diversity more compromised in more severe cases (Figure 3A), whereas in patients with CD, the diversity was more evenly distributed across the clinical scores (Figure 3B).

**Figure 4:**
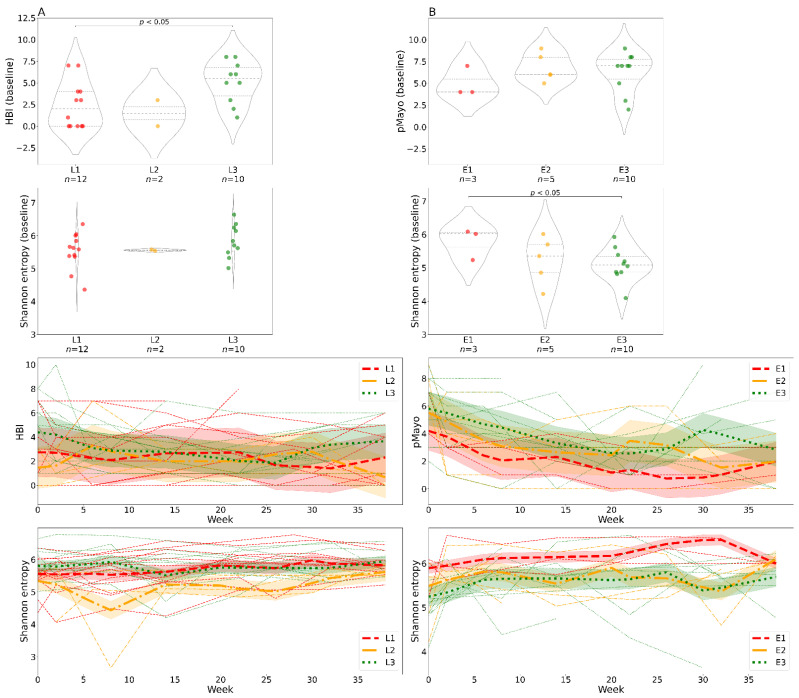
Differences in clinical scores and alpha diversity over time stratified by disease localization. In patients with Crohn’s disease (**A**) and ulcerative colitis (**B**). The violin plots show clinical scores and bacterial alpha diversity at baseline (week 0); the groups represent specific disease localizations. The line charts show changes in clinical scores and bacterial Shannon entropy over time and are color-coded by disease localization. Each line represents one individual; the bold lines represent locally weighted regression with a confidence interval fill. Abbreviations: HBI, Harvey–Bradshaw index; pMayo, partial Mayo score; L1, ileal Crohn’s disease; L2, colonic Crohn’s disease; L3, ileocolonic Crohn’s disease; E1, rectal ulcerative colitis; E2, ulcerative colitis distal to the splenic flexure; E3, ulcerative colitis affecting the whole large intestine and rectum; *n*, number of samples.

We examined the impact of disease localization on bacterial alpha diversity. Patients with CD with ileal involvement (L1) had significantly lower clinical scores at baseline than patients with ileocolonic involvement (L3), but Shannon entropy at baseline was similar in both groups (Figure 4A). Patients with UC with rectal localization (E1) showed the highest alpha diversity at baseline, which remained high throughout the whole course of the study (Figure 4B). This was also reflected in disease severity, with lower scores in patients with E1 localization. However, the differences were not significant. We found no significant differences in alpha diversity between baseline and endpoint for single localizations.

Our results confirmed that the drug choice was not random and that patients with UC with more severe disease were treated preferentially with IFX. Figure S4 illustrates the differences in clinical scores and bacterial diversity at baseline and during the therapy in both drug groups.

**Figure 5:**
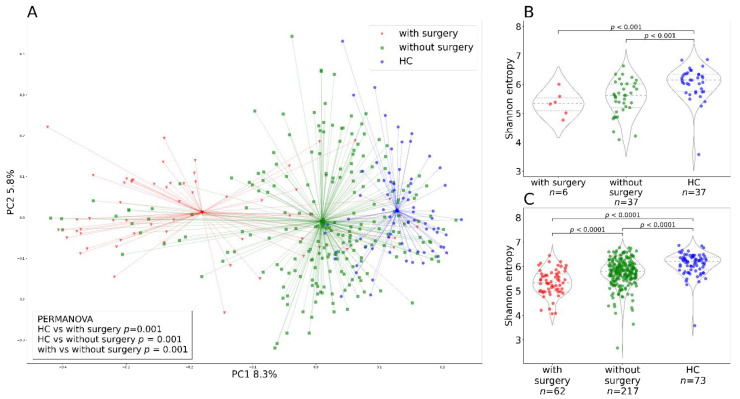
Alpha and beta diversity differences due to surgical procedure. (**A**) The PCoA plot shows beta diversity represented by the Bray–Curtis metric for patients with IBD with and without surgery, and healthy controls at various time points during the treatment including the baseline. Each dot represents a sample at a single time point and a line connects it to the cluster centroid. (**B**) Alpha diversity represented by Shannon entropy in Crohn’s disease patients at baseline categorized by intestinal surgery and in healthy controls. (**C**) Alpha diversity represented by Shannon entropy in the whole patients with IBD at various time points including baseline categorized by intestinal surgery and in healthy controls. Abbreviations: HC, healthy controls.

We also found that patients with CD who had undergone intestinal surgery (drainage, ileocecal resection) in the past showed a different beta diversity (Figure 5A), and their alpha diversity was diminished both at baseline (Figure 5B) and during the whole observation period (Figure 5C). No patients with UC had undergone surgery.

### 3.4. Changes of the Gut Bacteriome during Anti-TNF Therapy

Twenty-seven stool samples (17 CD, 10 UC) were obtained from patients at the endpoint of the study (week 38). We did not detect any changes in bacterial alpha diversity between week 0 (baseline) and week 38 in either patients with CD (Wilcoxon signed-rank test: *p* = 0.353) or patients with UC (Wilcoxon signed-rank test: *p* = 0.064) patients. In patients with UC, the difference between the baseline and the endpoint was larger than in patients with CD, but it was statistically insignificant (Figure S5). Thirteen taxa turned out to be differentially abundant between baseline and endpoint in patients with CD and 10 in patients with UC. Nevertheless, all of the differentially abundant taxa turned out to be structural zeros except for *Ruminococcus*, which was increased in patients with UC at the study endpoint (Table S1). The relative abundances of *Ruminococcus* during the observation period are shown in Figure S6.

**Figure 6:**
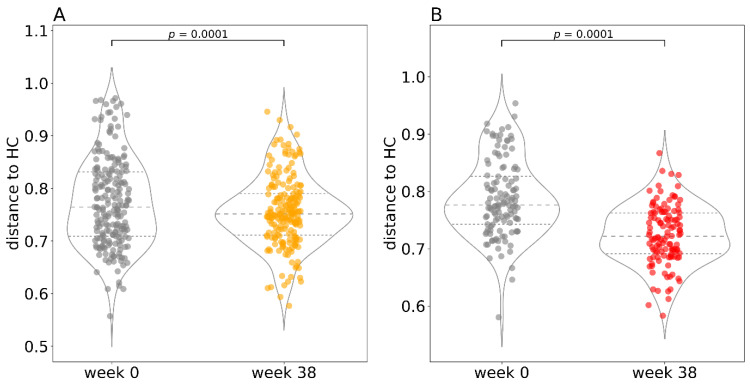
Distance to healthy controls at the beginning and at the end of therapy. A visual representation of the restricted permutation design with Bray–Curtis distance to healthy controls at the beginning (week 0) and at the end (week 38) of the study for Crohn’s disease (**A**) and ulcerative colitis (**B**) separately. In the violin plots, the middle dashed line represents the median and the outer dashed lines represent the first and third quartiles. Each dot in the superimposed strip plot represents a single distance from a patient at baseline (week 0) to a healthy control at baseline or a single distance from a patient at the endpoint (week 38) to a healthy control at the endpoint. Abbreviations: HC, healthy controls.

To evaluate changes in the gut bacteriome during anti-TNF therapy, we assessed the degree of beta diversity similarity to healthy controls (Figure 6). We calculated a Bray–Curtis distance matrix between all samples and then applied a restricted permutation test specifically designed to compare only the distances to HC at baseline (week 0) and at the endpoint (week 38). We discovered a significant difference between the baseline distance to HC and the endpoint distance to HC in both patients with CD and UC. However, due to the very low contribution of the diagnosis factor to the overall variance in the whole cohort (4%), we cannot rule out that these differences are caused by large dispersion of the measurements.

### 3.5. Metagenomic Predictions

**Figure 7:**
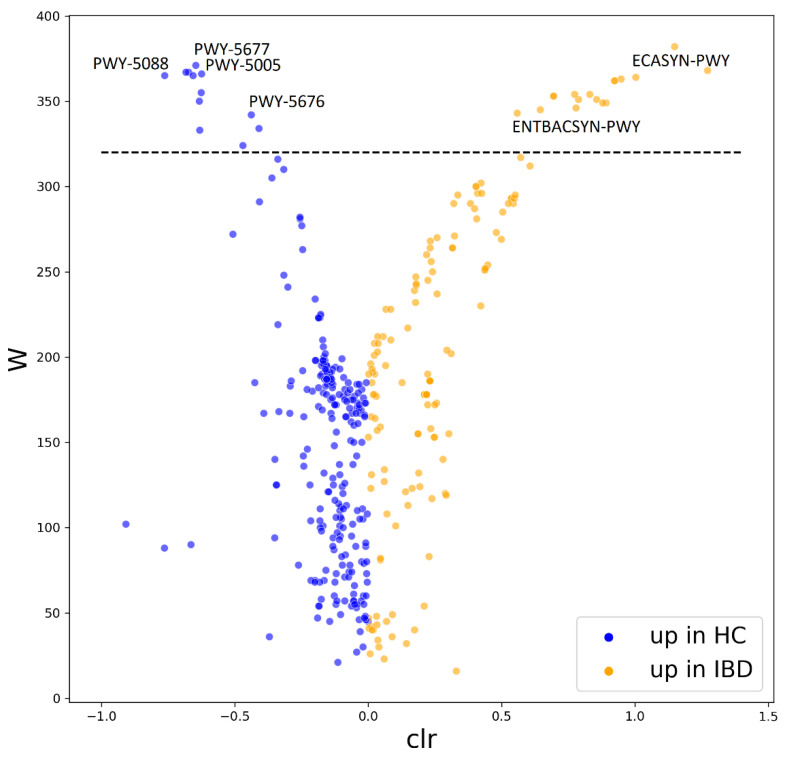
Differentially abundant pathways reconstructed from 16S rRNA data by PICRUST2. The volcano plot shows the differentially abundant pathways between healthy controls and patients with IBD as determined by ANCOM. Each dot represents a pathway entry from the MetaCyc database, which was reconstructed from 16S rRNA data. Pathways increased in HC are blue, pathways increased in IBD are orange. The dashed line indicates the W-statistic cut-off value. The full list of the differentially abundant features can be found in Table S2. Abbreviations: HC, healthy controls; IBD, patients with inflammatory bowel disease; W, W-statistic; clr, centered log ratio; PWY-5677, succinate fermentation to butanoate; PWY-5005, biotin biosynthesis; PWY-5088, L-glutamate degradation to propionate; PWY-5676, acetyl-CoA fermentation to butanoate; ECASYN-PWY, enterobacterial common antigen biosynthesis; ENTBACSYN-PWY, enterobactin biosynthesis.

Metabolic pathways from MetaCyc were reconstructed from our 16S rRNA data using PICRUST2 [31]. We identified a significant increase in multiple metabolic pathways in patients with IBD compared with HC (Figure 7, Table S2), further we describe only the most relevant to IBD pathology. The underrepresented metabolic pathways include fermentation to butyrate and propionate (PWY-5677, PWY-5088, PWY-5676). Further, biotin biosynthesis was decreased in patients with IBD (PWY-5005). Pathways including the biosynthesis of enterobactin, an iron chelator utilized by bacteria to acquire iron from the environment, and enterobacterial common antigen were increased in patients with IBD (ENTBACSYN-PWY, ECASYN-PWY).

### 3.6. Fungal Microbiome

We found no differences in alpha diversity represented by Shannon entropy between individuals in the healthy controls group (*p* = 0.327), probably due to the small sample size, because in the patient group the differences were significant (*p* = 0.009) between individuals (Figure S7). In terms of beta diversity calculated using Bray–Curtis dissimilarity, the individual factor explained only 5% of the variance in the HC group and 6% in the IBD group (Figure 1).

The community composition was dominated by the genera *Saccharomyces*, *Penicillium*, and *Candida* and the Ascomycota/Basidiomycota ratio was skewed towards Ascomycota in all three groups.

At baseline, the fungal community composition of patients with IBD was not significantly different from that of healthy controls. We did not find any significant changes in Shannon entropy over the course of therapy. Similarly, we observed no significant differences in Shannon entropy between the baseline and the endpoint of the study. Additionally, patients’ fungal communities were not shifted towards the HC mycobiome at the endpoint compared to the baseline (data not shown).

### 3.7. Serum Antibody Levels against Specific Commensals

Total levels of IgM, IgA, and IgG were measured in the serum of HC and patients with IBD at baseline (week 0) and at the endpoint (week 38; Table 2).

**Figure 8:**
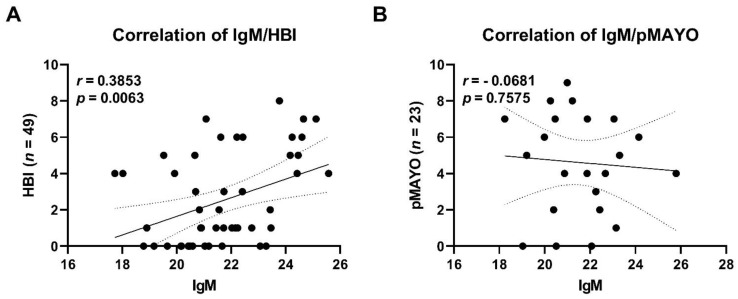
Correlation of total IgM serum levels and disease severity in patients with Crohn’s disease (**A**) and patients with ulcerative colitis (**B**). Each dot represents a sample from a patient at a specific time point. Trendline (bold line) including confidence interval (dotted line) is shown. Abbreviations: HBI, Harvey–Bradshaw index; pMayo, partial Mayo score; *n,* number of samples.

Differences in the total levels of IgM, IgA, and IgG were significantly different between HC and patients with CD both at baseline and at the endpoint. Pairwise differences between the baseline and the endpoint in patients with CD were significant only for IgM. A positive correlation between total serum IgM levels and disease severity was found in patients with CD (Figure 8).

We measured specific IgM targeted against antigenic structures of assorted bacteria (*Lactobacillus plantarum*, *Bifidobacterium adolescentis*, *Blautia coccoides*, *Roseburia intestinalis*, *Faecalibacterium prausnitzii*, *Bacteroides thetaiotaomicron*, *Escherichia coli*, *Prevotella ruminicola*, *Ruminococcus flavefaciens*, and *Eubacterium rectale*). We detected a significant increase in antibodies against most of the bacteria (except *F. prausnitzii*) between the baseline and the endpoint of the therapy in patients with CD (Table 3). Conversely, in patients with UC none of the specific antibodies were significantly different between the baseline and the endpoint. We did not detect any significant differences between specific antibody levels of healthy controls and patients with IBD.

### 3.8. Patients’ PBMCs Produce Increased Levels of IL-17 at the Endpoint of Therapy

Since the immune response to microbiota might change during anti-TNF therapy, we analyzed the T cell response to microbial stimuli before the start of the therapy (week 0) and after 38 weeks. We isolated PBMCs and challenged them in vitro with a panel of gut commensals and measured the levels of IL-17A produced. We found that at week 38 T cells from patients with UC react with increased IL-17 production to antigens from *Blautia* (Figure S8A). Additionally, PBMCs isolated from patients with CD react more strongly to antigens isolated from *E. coli* K6 at week 38 (Figure S8B).

## 4. Discussion

Monitoring the microbiome during anti-TNF therapy provided interesting insights into the link between gut microbial composition and disease advancement and severity.

Since data on microbiome temporal variability are scarce in the literature, we conducted a longitudinal analysis in a HC group to provide a reference for our IBD patient study group. The fecal microbiome of HC was relatively stable in time and did not show any significant temporal fluctuations (Figure S2), in agreement with previous studies [35,36]. We confirmed that individual differences play a huge role in both alpha and beta diversity (Figure S1, Figure 1) [37]. Interindividual variation exceeds temporal variation. In our cohort, almost 50% of the Bray–Curtis bacterial beta diversity variance was explained by the individual factor, whereas the shared variance of the cohort in time contributed only 2% and individual variability in time by less than 26%. Unsurprisingly, BMI also substantially contributed (17%) to the overall bacterial beta diversity variability in our HC cohort, which is in line with other studies [38].

There is quite robust evidence that patients with IBD differ from HC in their gut microbiome diversity and composition. Moreover, it appears the two distinct subtypes of IBD have their specific fecal microbiome signature [25]. However, it is not clear whether these microbiota changes are the cause or a consequence of intestinal inflammation. In agreement with the existing literature, we found both alpha and beta diversity differences between HC and patients with IBD (Figure 2). In addition, we also found microbiome diversity differences between patients with CD and UC, where Shannon entropy was lower in the patient group with UC. This might be due to the large proportion of patients with CD in remission (65%), who started the treatment to maintain remission, in contrast to no patients with UC in remission entering the study. Different results are reported by Pascal et al. and Ventin-Holmberg [25,39], who found patients with CD to have the lowest alpha diversity, and by Nishino et al. [24], who did not find any significant differences between the two disease types. As previously mentioned, this discrepancy could be attributed to the disease severity and disease localization of the participating individuals in the Nishino study (49% and 73% of patients in remission for UC and CD, respectively) and the Ventin-Holmberg study (66% and 57% of patients in remission for UC and CD, respectively).

During the course of therapy, more pronounced, although statistically insignificant, changes in terms of disease severity and alpha diversity were observed in patients with UC compared with patients with CD (Figure S3). This outcome could reflect the aforementioned high percentage of patients with CD in remission entering the study. Disease severity negatively correlates with bacterial alpha diversity in patients with UC; the higher the pMayo score, the lower the bacterial diversity (Figure 3). The anti-TNF therapy acts to reduce the gut inflammation and as our results suggest it improves alpha diversity, which in turn regulates the gut environment. The resulting effect of the therapy and changes in microbiome could promote gut tissue healing.

Patients with UC with disease localized to the rectum had the highest diversity and the lowest clinical scores, which can be explained by the relatively low bacterial load in the rectum in comparison to the colon where most fermentation processes take place (Figure 4) [40]. Shannon entropy was similar in patients with CD with different disease localizations, but the clinical scores differed significantly between patients with ileal (L1) and ileocolonic (L3) disease localization, indicating that patients with a larger affected area suffered from a more severe disease. The reason why we observe less pronounced differences in fecal bacterial diversity in patients with CD with different gut region involvement could be that fecal samples represent mainly the colonic rather than ileal microenvironment [41].

Discussion on the differences between the efficacy of chimeric infliximab and fully human adalimumab is still not settled in the medical literature. This is largely because of a lack of clinical trials, which are challenging to design due to different dosing regimens, disease types and disease activity in the patients involved [42]. Although some studies claim that the effects of these drugs are similar [43,44], others suggest that IFX and ADA should be used under different circumstances [45]. Therefore, the drug choice is dependent on the doctor’s judgment and local clinical guidelines, including previous treatment success or failure, adverse events, disease subtype, severity, and localization, as well as local availability, which precludes randomization of the study population. In our study cohort, we found that patients with UC treated with IFX had higher disease scores at baseline and during therapy (Figure S4), which is due to the clinical guidelines assigning specific patients to a specific treatment.

Another factor that turned out to play a role in bacterial diversity was previous intestinal surgery (Figure 5). Patients who underwent a surgical procedure (drainage or ileocecal resection) tended to have a lower bacterial diversity. Although the presence or absence of intestinal surgery accounted for only 3% of the beta diversity variation, patients could be clustered based on the surgical procedure. This could be explained by the fact that patients who had undergone intestinal surgery had very severe disease, which is accompanied by more extreme dysbiosis. Additionally, antibiotics used for bowel preparation before surgery can induce dysbiosis, which can persist more than 3 months after administration [46]. Lastly, the tissue stress and change in gut physiology can lead to altered food digestion and absorption which can contribute to the observed dysbiosis [47].

A unique feature of our study was the longitudinal design, where we tracked the patients with IBD over the course of therapy from week 0 to week 38, collecting as many as 8 samples per individual. We did not find any associations between the treatment outcome and the changes of the microbiome in time. Pairwise comparisons between the baseline (week 0) and the endpoint (week 38) showed no significant differences in diversity neither in patients with CD nor in patients with UC (Figure S5). Other studies reported similar results [15,48,49]. To our knowledge, only one study [16] showed a significant increase in alpha diversity post-treatment in 18 patients with severe CD, 88% of whom reached remission.

Contradictory data on the effect of *Ruminococcus* exist in the current literature. Ruminococci comprise multiple phylogenetically distinct bacterial genera, which are part of the healthy human microbiome, but some are enriched in IBD flares. Although ruminococci from the Lachnospiraceae family such as *R. gnavus* and *R. torques* are associated with patients with IBD [50,51] and are reported to produce inflammatory polysaccharides [52], the true ruminococci from Ruminococcaceae family are enriched in HC [53,54] or associated with IFX responders [39,55]. Ruminococci are well adapted for survival in the environment of elevated oxidative stress that exists in gut of patients with IBD through adhesion and degradation of protective intestinal mucins [51], but under different conditions can also metabolize available substrates to produce beneficial SCFAs [56,57]. Here, we showed that uncultured *Ruminococcus* from the family Ruminococcaceae was increased after anti-TNF therapy in patients with UC (Table S1, Figure S6).

A shift of the bacterial community composition in patients with IBD towards that of HC is generally considered a sign of microbiome improvement. We noted a significant shift of beta diversity similarity towards HC in both patients with CD and UC at the study endpoint, suggesting a change towards a healthy microbiome (Figure 6). The same results were obtained by Aden et al., who also reported a shift towards healthy subjects in IBD [15]. This result is congruent with our data on alpha diversity where more dramatic changes occurred in the patient group with UC as well.

Metagenomic predictions revealed several known pathways lacking in patients with IBD compared with healthy individuals (Figure 7, Table S2). The first being the SCFAs, butyrate and propionate, metabolites produced by the gut microbiota, which are known for their immunomodulatory properties and serve as an energy source for colonocytes [58,59]. The second pathway identified as underrepresented in the microbiome of patients with IBD was biotin biosynthesis. Biotin is a vitamin, which can be synthesized by the gut microbiota and instantly absorbed directly in the large intestine. Biotin and other micronutrients deficiency have been described in patients with IBD [60]. Recent studies report that mice deprived of biotin show signs of the leaky gut syndrome, including the downregulation of tight junction proteins typical for patients with IBD [61,62]. A decreased biosynthesis of biotin by the intestinal bacteria could contribute to the reduced availability of this vitamin in the gut of patients with IBD. We also identified pathways that were overrepresented in patients with IBD compared with HC (Figure 7, Table S2). Increased biosynthesis of enterobactin was predicted in the microbiome of patients with IBD, which suggests possible colonization by adherent invasive *Escherichia coli* (AIEC) capable of producing enterobactin. Enterobactin is an iron-chelating molecule that helps pathogens to overcome low iron availability [63]. Furthermore, we detected an increased biosynthesis of enterobacterial common antigen (ECA) in patients with IBD, suggesting the presence of members of this family with pathological potential. ECA is an antigen found on the surface of microbes in the family Enterobacteriaceae. In a complex with lipopolysaccharide, it can elicit an unwanted immune response in the host [64]. Pioneering studies in the 1970s first observed immune response to ECA in patients with UC [65,66]. Nonetheless, the metagenomic analysis only extrapolates from 16S rRNA data and a more in-depth analysis is needed to confirm these findings.

Fungi represent a minor fraction of the gut microbiome compared to bacteria. Similar to bacteria, there is quite substantial interindividual variability between individual mycobiomes, which accounted for 25% of the beta diversity variance in our patient cohort (Figure S7, Figure 1) This finding is in accord with a study of the fungal microbiome in healthy individuals [21]. We have not detected any differences in the fungal microbiome between the diagnostic groups, nor did we detect any temporal changes during the therapy. This is in contrast to a study with 235 patients with IBD where alpha diversity was decreased in patients with UC and the community composition differed between patients of both IBD diagnoses and HC [20]. Nevertheless, the root of the inconsistency may be in the smaller sample size of our IBD cohort in comparison to the Sokol’s study comprising almost five times more patients. The genera *Saccharomyces*, *Penicillium*, and *Candida* were found to be dominant in almost all samples, but some samples had a quite high amount of unassigned ASVs, not uncommon in fungal studies [21]. Importantly, fungi detected in stool can be just transient visitors of the gut [67] and heavily depend on diet composition [68,69] hence masking the effect of the treatment.

Total levels of IgM, IgG, and IgA were increased in patients with IBD compared to healthy controls (Table 2), suggesting an ongoing inflammation in these subjects and the presence of immunoglobulins directed against specific bacterial species. One surprising finding is that patients with CD had increased levels of IgM at the study endpoint compared with the baseline. At the endpoint we counted 77% of patients in remission, so the explanation may lie in the protective role of IgM, which has been shown to be beneficial in patients with sepsis [70]. Given the fact that B cells can produce and react to TNF, the authors speculate that blockade of TNF leads to increased number of B cells and enhanced production of antibodies as already documented in rheumatoid arthritis patients [71]. The increase of the IgM but not IgG or IgA at the study endpoint could be explained by increased amount of memory pre-switch B cells in patients with CD [72].

While the presence of antibodies against *E. coli* antigens in patients with IBD has been established for decades [73], we wanted to expand the scope of antigens and tested the humoral response to a whole panel of gut commensals (Table 3). We found an increased IgM response at week 38 in patients with CD against all antigens tested except *Faecalibacterium prausnitzii*, which has been repeatedly linked with HC or patients who achieved remission [74]. A recent study on bacterial flagellins found increased levels of IgG and IgA antibodies against members of the *Lachnospiraceae* family, including *Roseburia* and *Eubacterium* (also present in our panel), in sera of patients with CD. These were linked with elevated specific memory T cells and disease complications [75]. Taking into account recent studies identifying antibody levels against specific commensals in healthy controls [76,77], we speculate that the absence of significant differences in antibody levels between HC and IBD can be caused by this peculiarity.

Accumulating evidence suggests that the role of TNF-α and IL-17 in IBD pathogenesis is more complicated than previously thought since these cytokines can exert both pro- and anti-inflammatory effects [78,79]. They can promote intestinal barrier repair and several mouse studies confirmed that they might suppress intestinal inflammation [80,81]. In addition, anti-IL-17 therapy has not shown therapeutic efficacy in the treatment of patients with CD [82]. Besides, new cases of IBD have been reported in patients receiving anti-IL-17 therapy to treat their dermatological and rheumatic diseases [83]. Thus, the intriguing question is how anti-TNF treatment affects IL-17 production. In our study, we have shown that blood cells isolated after 38 weeks of therapy respond with increased IL-17 production to specific bacterial antigens (Figure S8). This finding is consistent with a study that found increased serum production of IL-17 in patients receiving anti-TNF treatment [84]. Similarly, serum IL-17 levels of patients with CD with active disease were reported to be lower than those of healthy controls and patients with CD in remission [85]. The importance of IL-17 production in IBD could be also in its influence on the induction of anti-microbial peptides and consequent maintenance of gut homeostasis [86]. Future microbiome studies would benefit from targeting a very particular patient group within one diagnosis, defined disease localization and treatment to eliminate as many variables influencing microbial diversity as possible. These variables and not so large sample size were the main limitations of our study which made the results challenging to interpret.

## 5. Conclusions

In conclusion, we confirmed differences in the bacterial microbiome of HC and patients with IBD and also between patients with CD and UC. These results are complemented by metabolic pathways predictions, which also differed between HC and patients with IBD. Moreover, we highlight that interindividual variability in bacteriome diversity plays a greater role than temporal variability and disease phenotype. This might explain why studies consistently report changes in diversity, but evidence for differential abundance has been mixed. In addition, we demonstrated the importance of disease severity, disease localization, and intestinal surgical interventions in bacterial microbiome research suggesting the study cohort composition has tremendous impact in the observed diversity. We showed that at the study endpoint, beta diversity of both patients with CD and UC were more similar to HC subjects than at baseline, although we have identified only *Ruminococcus* to be differentially abundant in patients with UC. Furthermore, we detected an increased humoral response to multiple gut commensals in patients with CD at the study endpoint. These results emphasize the importance of gut microbiota diversity and composition as well as specific immune response to gut bacteria in IBD pathogenesis. TNF inhibitor therapy influences these factors to a different extent in patients with CD and UC.

## Figures and Tables

**Table 1:** Comparison of anthropometric and clinical parameters in healthy controls and patients with IBD included in the gut microbiome analysis at baseline (week 0) and at the endpoint (week 38). Multiple comparisons between groups were evaluated by the Kruskal–Wallis test followed by Dunn’s multiple comparison test. Changes during anti-TNF therapy (week 0 vs. week 38) were evaluated by Wilcoxon matched pairs signed-rank test. Medians are reported with the first and third quartiles in parentheses. Abbreviations: CD, Crohn’s disease; UC, ulcerative colitis; HC, healthy control; BMI, body mass index; HBI, Harvey–Bradshaw index; pMayo, partial Mayo score; CRP, C-reactive protein; FC, fecal calprotectin; Hb, hemoglobin; PLT, platelet count; WBC, white blood cells; *n*, number of participants.

	HC (*n* = 37)	CD (*n* = 17) Week 0	CD (*n* = 17) Week 38	∆ CD (*p*-Value)	CD vs. HC (*p*-Value)	UC (*n* = 10) Week 0	UC (*n* = 10) Week 38	∆ UC (*p*-Value)	UC vs. HC (*p*-Value)
Male: female	15:22	5:12			0.8042	2:8		-	0.8684
Age	36.5 (28.8, 41.3)	35.0 (26.5, 44.0)		-	0.7800	31.0 (26.0, 41.3)		-	0.4228
BMI	21.8 (20.5, 26.2)	22.2 (20.8, 26.9)		-	0.8274	21.3 (19.2, 24.4)		-	0.1901
Disease duration in years	-	4.0 (2.0, 13.5)		-	-	2.5 (1.0, 7.5)		-	-
Age at diagnosis	-	26 (22.5, 35)		-	-	27 (21.5, 31)		-	-
Remitters	-	11/17	13/17	-	-	0/10	5/10	-	-
HBI/pMayo	-	3 (0, 6)	1 (0.5, 5)	0.3931	-	7 (3.8, 8.3)	2.5 (0, 4.3)	0.0039	-
CRP (mg/L)	0.9 (0.4, 1.9)	4.2 (1.0, 8.9)	1.3 (0.5, 5)	0.0225	0.0037	4.1 (0.9, 22.3)	0.95 (0.6, 2.8)	0.1641	0.0124
WBC	5.9 (4.9, 6.9)	8.0 (6.2, 11.0)	5.9 (5, 7.8)	0.0089	0.0135	10.4 (6.1, 12.3)	5.9 (4.4, 7.3)	0.0371	0.0071
PLT	247 (218, 272)	328 (269, 416)	283 (220, 388)	0.0697	0.0007	276 (251, 387)	278 (222, 310)	0.4160	0.1136
Ferritin (µg/L)	NA	48.1 (19.8, 80.0)	30.9 (12.3, 56.8)	0.3778	NA	28.0 (11.7, 52.2)	41.0 (9.5, 78.6)	0.9219	NA
Hb (g/L)	141 (134, 158)	130 (123, 150)	134 (127, 144)	0.4411	0.0495	128 (109, 1367)	134 (121, 146)	0.0918	0.0067
FC (μg/g)	NA	407 (148, 1028)	162 (38, 284)	0.0181	NA	661 (424, 2838)	229 (59, 828)	0.0039	NA

**Table 2:** Total serum IgM, IgA, and IgG levels in healthy controls and patients with IBD. The antibody levels (mg/mL) of patients with IBD at week 0 and 38 and of healthy controls are reported as medians with first and third quartiles in parentheses. Data were log-transformed and multiple comparisons between patients and healthy controls were evaluated by one-way ANOVA followed by Tukey’s multiple comparison test. The comparisons between antibody levels in patients at week 0 and week 38 were evaluated by paired *t*-tests. Abbreviations: ns, not significant; CD, Crohn’s disease; UC, ulcerative colitis; HC, healthy controls.

		Week 0	Week 38	HC	Week 0 vs. HC	Week 38 vs. HC	Week 0 vs. Week 38
IgM	CD	2.1 (1.3; 3.4)	2.4 (1.4; 7.6)	1.1 (0.8; 1.8)	0.020	<0.001	<0.001
	UC	2.6 (0.9; 4.0)	1.3 (1.0; 3.2)		ns	ns	ns
IgA	CD	1.9 (0.5; 5.0)	2.2 (0.6; 6.6)	0.7 (0.5; 1.4)	0.009	0.010	ns
	UC	2.5 (1.3; 4.4)	3.2 (1.3; 5.7)		0.039	0.024	ns
IgG	CD	15.9 (8.1; 35.1)	11.2 (5.3; 20.4)	1.1 (0.8; 1.8)	<0.001	0.002	ns
	UC	10.5 (6.5; 18.1)	10.6 (5.7; 59.8)		0.019	<0.001	ns

**Table 3:** Comparison of specific IgM levels in healthy controls and patients with inflammatory bowel disease (IBD). Levels of IgM specifically targeted to assorted bacterial lysates in patients with IBD at week 0 (w0) and 38 (w38), and in healthy controls. Data were log-transformed and multiple comparisons between patients and healthy controls were evaluated by one-way ANOVA followed by Tukey´s multiple comparison test. The comparisons between antibody levels in patients at week 0 (w0) and week 38 (w38) were evaluated by paired *t*-tests. Arrows indicate increase of the antibody levels. Abbreviations: ns, not significant; CD, Crohn’s disease; UC, ulcerative colitis; HC, healthy controls.

	CD w0 vs. HC	CD w38 vs. HC	CD w0 vs. w38	UC w0 vs. HC	UC w38 vs. HC	UC w0 vs. w38
*Lactobacillus*	ns	ns	↗ *p* = 0.005	ns	ns	ns
*Bifidobacterium*	ns	ns	↗ *p* = 0.010	ns	ns	ns
*Blautia*	ns	ns	↗ *p* = 0.005	ns	ns	ns
*Roseburia*	ns	ns	↗ *p* < 0.001	ns	ns	ns
*Faecalibacterium*	ns	ns	ns	ns	ns	ns
*Bacteroides*	ns	ns	↗ *p* = 0.005	ns	ns	ns
*Escherichia*	ns	ns	↗ *p* = 0.003	ns	ns	ns
*Prevotella*	ns	ns	↗ *p* = 0.005	ns	ns	ns
*Ruminococcus*	ns	ns	↗ *p* = 0.001	ns	ns	ns
*Eubacterium*	ns	ns	↗ *p* < 0.001	ns	ns	ns

## Data Availability

Data from amplicon sequencing are available in the Sequence Read Achieve with a BioProject ID: PRJNA757573. ASV tables and taxonomy files including the notebooks with downstream data analysis can be found in GitHub repository https://github.com/dagmarschierova/anti-TNF.

## Supplementary Materials

The following are available online at https://www.mdpi.com/article/10.3390/cells10113188/s1, Figure S1: Interindividual variation in the bacterial microbiome of patients with Crohn’s disease, ulcerative colitis and healthy controls, Figure S2: Changes in bacterial alpha diversity of the healthy control cohort over time, Figure S3: Changes in bacterial alpha diversity of patients with inflammatory bowel diseases over time, Figure S4: Differences in clinical scores and alpha diversity over time stratified by the administered drug in patients with inflammatory bowel diseases, Figure S5: Bacterial alpha diversity differences between the baseline and the endpoint for patients with Crohn’s disease, ulcerative colitis, and healthy controls, Figure S6: Relative abundance of uncultured *Ruminococcus* during the study period in patients with inflammatory bowel diseases, Figure S7: Interindividual variation in the fungal community of patients with Crohn´s disease and ulcerative colitis, Figure S8: IL-17 production by PBMCs isolated from patients with Crohn’s disease and ulcerative colitis at week 0 and week 38, Table S1: Differentially abundant taxa in patients with Crohn´s disease and ulcerative colitis between the baseline and the endpoint, Table S2: Differentially abundant metabolic pathways reconstructed from 16S data by PICRUST2 between healthy controls and patients with inflammatory bowel diseases.
