# Supplementary material for: Fecal Microbiome Changes and Specific Anti-Bacterial Response in Patients with IBD during Anti-TNF Therapy
Source: Cells. 2021 Nov 16;10(11):3188. doi: 10.3390/cells10113188 (PMC8617723; doi:10.3390/cells10113188)
Supplement: Supplementary file 1 [file cells-10-03188-s001.zip › cells-1454183-supplementary.pdf]

## Supplementary material

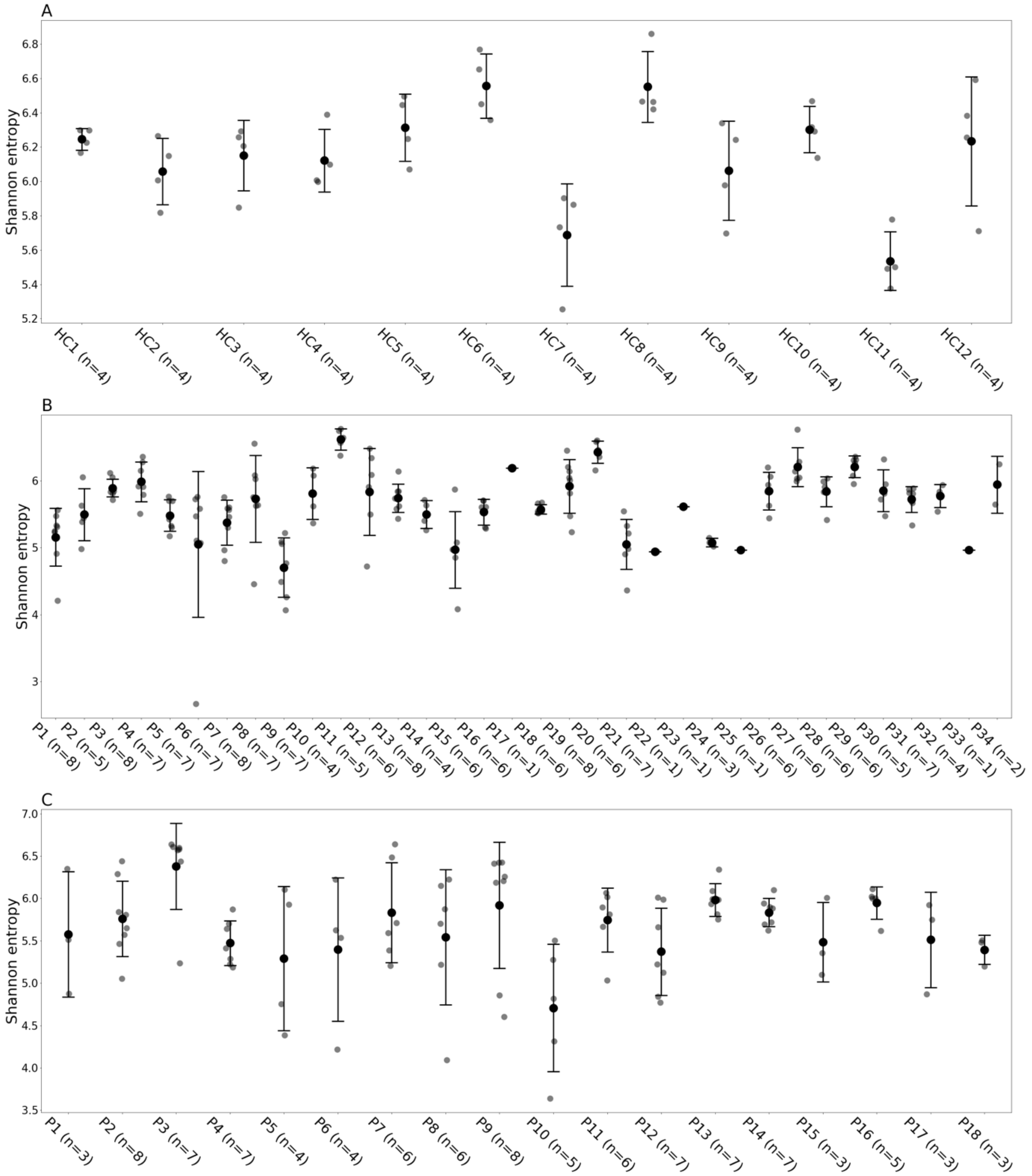


**Figure S1: Interindividual variation in the bacterial microbiome of patients with Crohn´s disease, ulcerative colitis and healthy controls.** Alpha diversity represented by Shannon entropy is plotted for healthy controls (**A**), patients with Crohn´s disease (**B**), and ulcerative colitis (**C**) at several time points during the treatment including baseline. Repeated measurements are shown for each individual along with the mean and standard deviation. Significant difference between individuals was determined by the Kruskal-Wallis test p=0.001 for HC, p<0.001 for IBD patients, IBD (inflammatory bowel disease), CD (Crohn´s disease), UC (ulcerative colitis), n (number of samples)

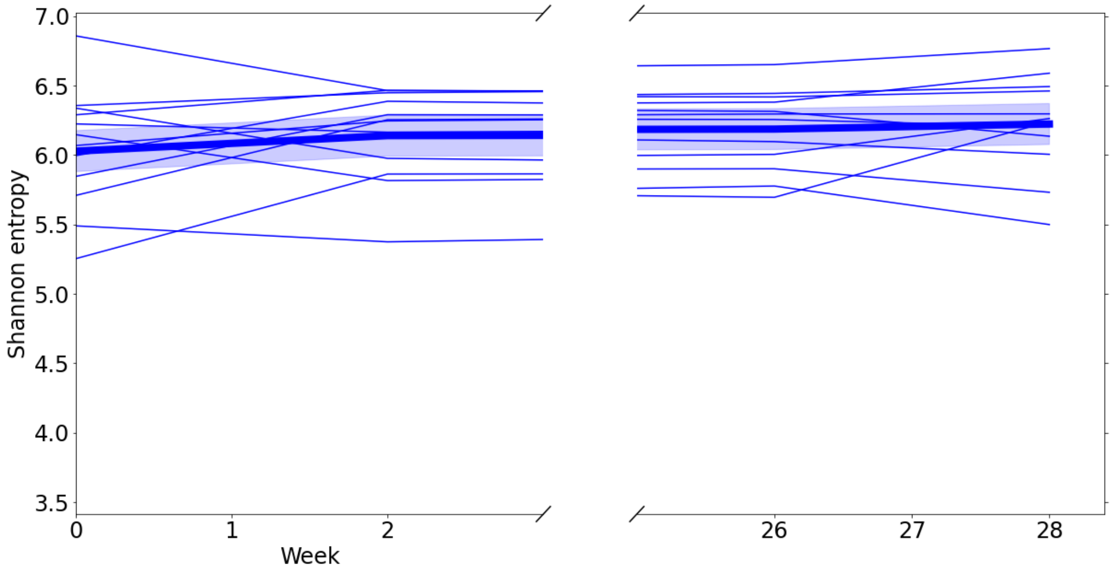


**Figure S2: Changes in bacterial alpha diversity of the healthy control cohort over time.** A longitudinal view of the alpha diversity in healthy controls (12 individuals). Shannon entropy is plotted at 4 different time points (week 0, 2, 26, 28). Each line represents one individual, the bold lines represent locally weighted regression with a confidence interval fill.

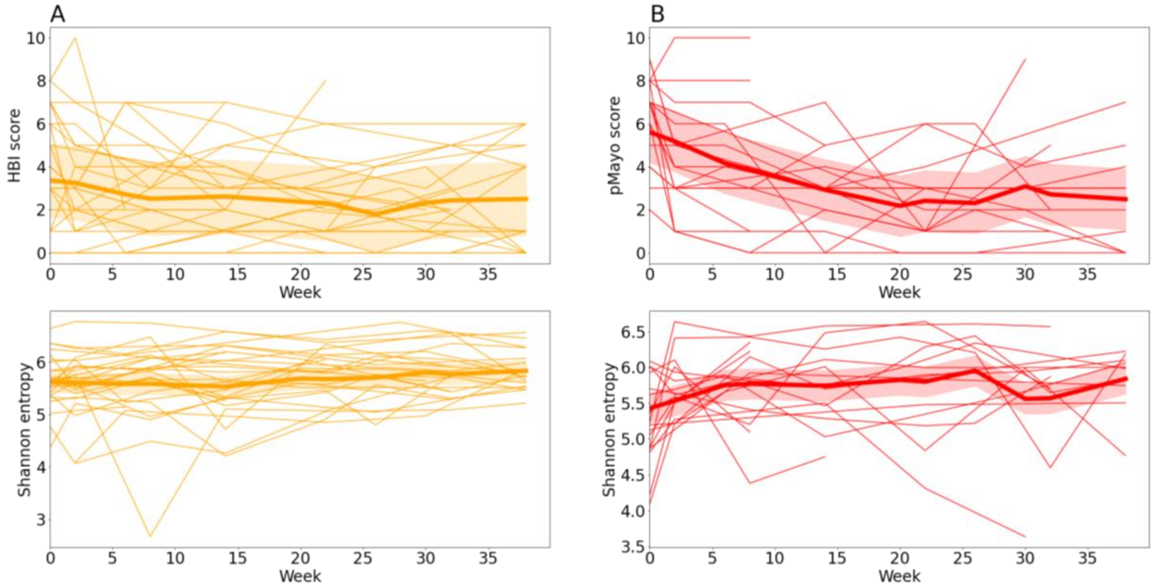


**Figure S3: Changes in bacterial alpha diversity of patients with inflammatory bowel diseases over time.** A longitudinal view of the entire patient cohort displayed for **(A)** Crohn's disease (34 patients) and **(B)** ulcerative colitis (18 patients) separately. Changes in clinical score and Shannon entropy are plotted over time. Each line represents one individual, the bold lines represent locally weighted regression with a confidence interval fill. IBD (inflammatory bowel disease)

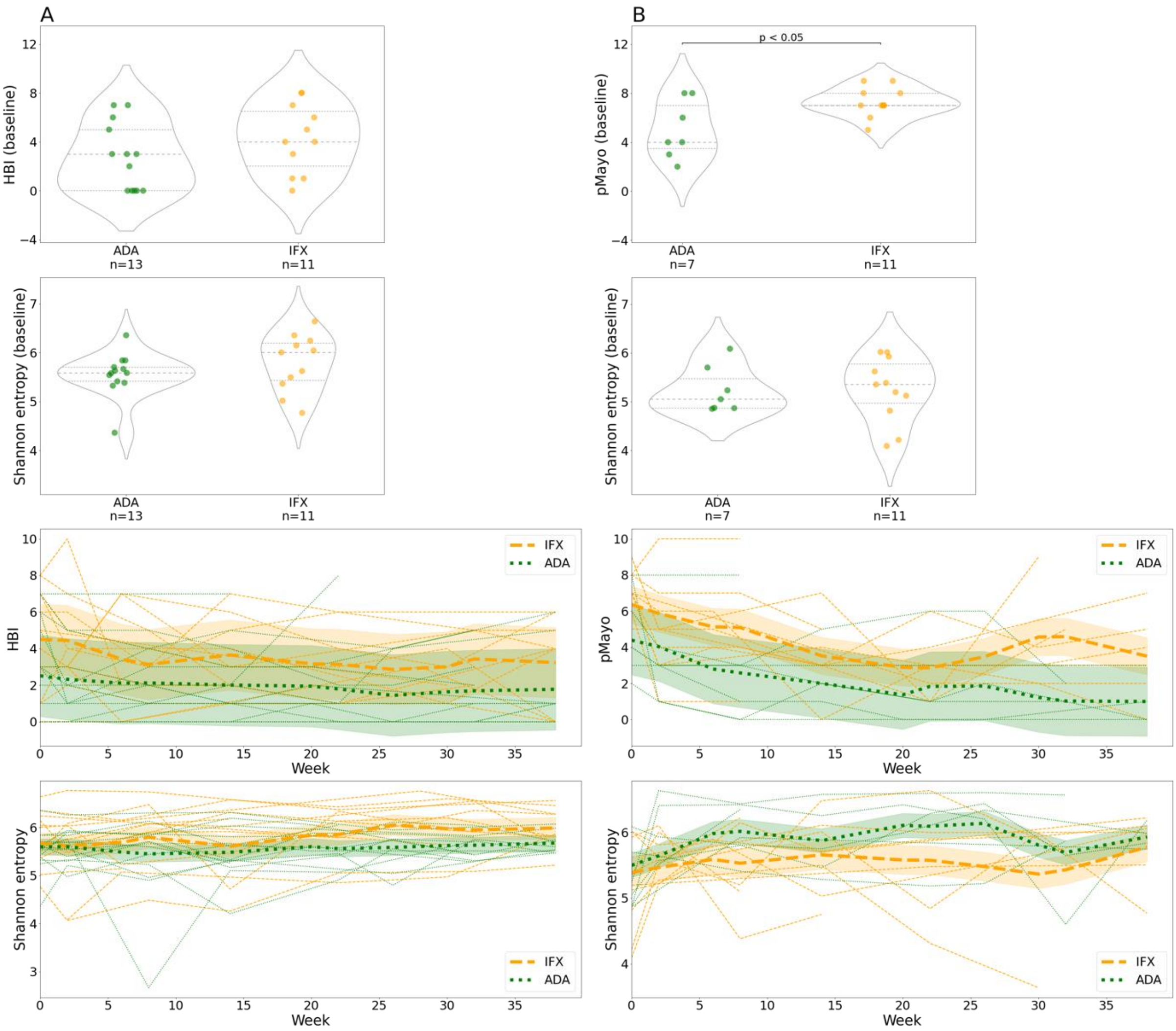


**Figure S4: Differences in clinical scores and alpha diversity over time stratified by the administered drug in patients with inflammatory bowel diseases**. In column **(A)** data for Crohn's disease is displayed, in column **(B)** data for ulcerative colitis is displayed. The violin plots show clinical scores and alpha diversity at baseline (week 0). The line charts show changes in clinical scores and Shannon entropy over time and are color-coded by the administered drug. Each line represents one individual, the bold lines represent locally weighted regression with a confidence interval fill. (HBI) Harvey-Bradshaw Index, (pMayo) partial Mayo score, IFX (infliximab), ADA (adalimumab), n (number of samples), IBD (inflammatory bowel disease)

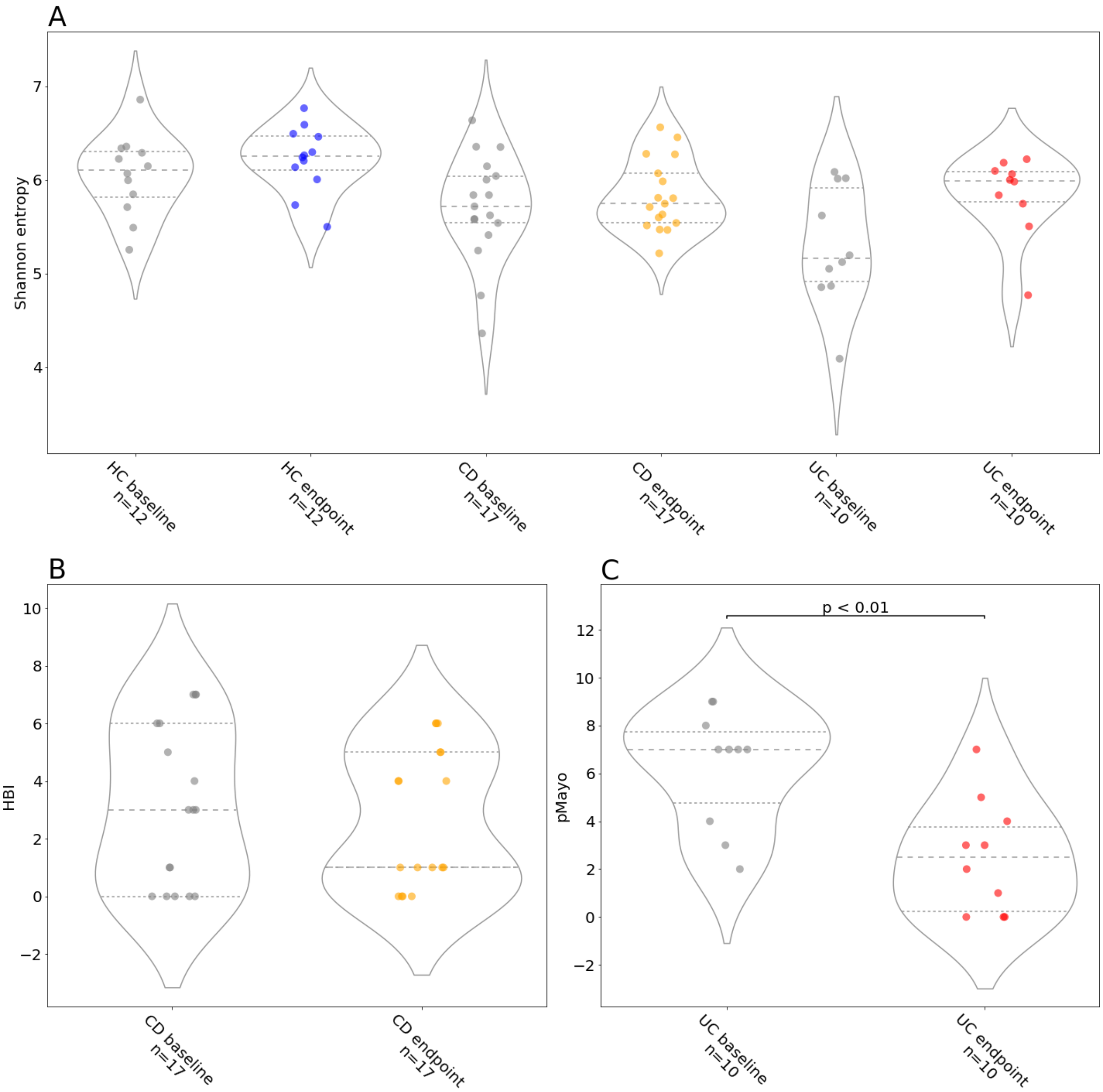


**Figure S5: Bacterial alpha diversity and clinical scores differences between the baseline and the endpoint for patients with Crohn´s disease, ulcerative colitis, and healthy controls.** A visual representation of Shannon entropy differences (**A**) between baseline (week 0) and endpoint (week 38) for Crohn's disease patients, ulcerative colitis patients and healthy controls. Disease severity between baseline and endpoint is shown separately for Crohn's disease patients (**B**) and ulcerative colitis patients (**C**). In the violin plots, the middle dashed line represents the median and the outer dashed lines represent the first and third quartiles. HC (healthy controls), CD (Crohn's disease), UC (ulcerative colitis), n (number of samples), HBI (Harvey-Bradshaw Index), pMayo (partial Mayo score).

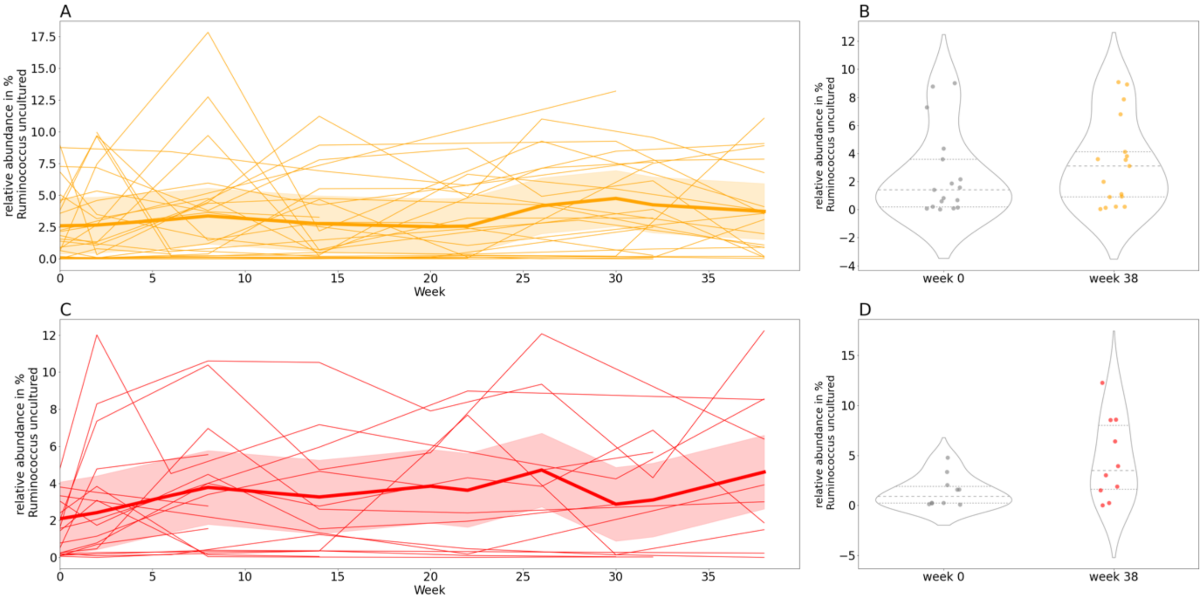


**Figure S6: Relative abundance of uncultured *Ruminococcus* during the study period in patients with inflammatory bowel diseases**. A longitudinal overview of the *Ruminococcus* relative abundance for patients with **(A, B)** Crohn's disease (34 patients) and **(C, D)** ulcerative colitis (18 patients) separately. Each line represents one individual, the bold lines represent locally weighted regression with a confidence interval fill. The violin plots show relative abundance of uncultured *Ruminococcus* at baseline (week 0) and at the study endpoint (week 38).

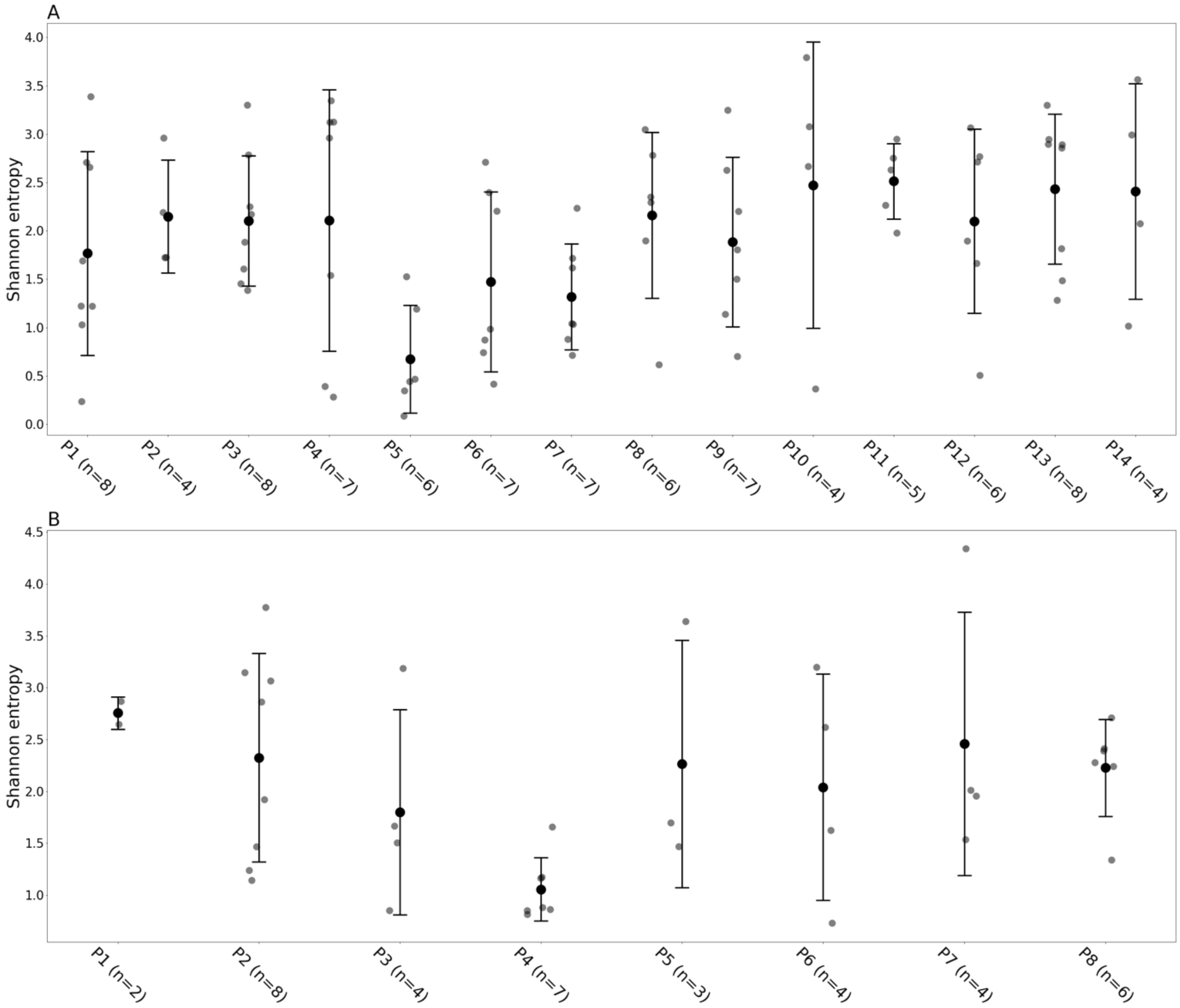


**Figure S7: Interindividual variation in the fungal community of patients with Crohn´s disease and ulcerative colitis.** Alpha diversity represented by Shannon entropy is plotted for patients with Crohn´s disease (**A**) and ulcerative colitis (**B**) at several time points during the treatment including baseline. Repeated measurements are shown for each individual along with the mean and standard deviation. n (number of samples).

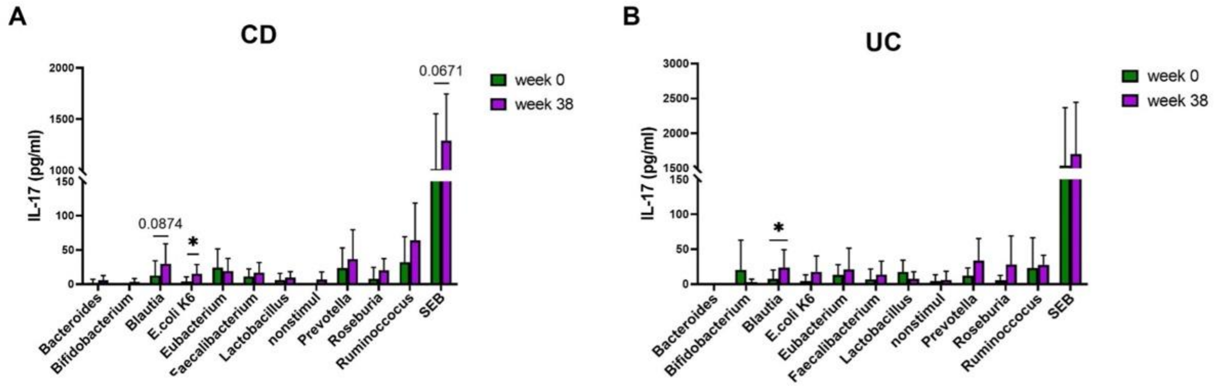


**Figure S8: IL-17 production by PBMCs isolated from patients with Crohn´s disease and ulcerative colitis at week 0 and week 38.** PBMC from CD **(A)** or UC **(B)** produce higher levels of IL-17 at week 38 after 72hod of stimulation with specific bacterial antigens as determined by the Mann–Whitney test. *$p<0.05$, UC (n = 5), CD (n = 8), SEB (Staphylococcal enterotoxin B), a polyclonal T cell activator was used as a positive control, CD (Crohn's disease), UC (ulcerative colitis)

**Table S1: Differentially abundant taxa in patients with Crohn´s disease and ulcerative between the baseline and the endpoint of the study**. The table shows differentially abundant taxa determined by ANCOM2.1 between the baseline (week 0) and the endpoint (week 38) of the study. W-statistics and centered log ratios (clr) for both diagnostics groups are shown. $W_{max}$ was 103 for CD patients, $W_{max}$ was 87 for UC patients and a cut off 0.8W was chosen. CD (Crohn's disease), UC (ulcerative colitis), (str. zero) structural zero

| **CD** | | | **UC** | | |
|---|---|---|---|---|---|
| **taxa detected cut off 0.8W** | **W** | **clr** | **taxa detected cut off 0.8W** | **W** | **clr** |
| *Bifidobacterium animalis* | str. zero | 0.2 | *Paraprevotella uncultured bacterium* | str. zero | -0.7 |
| *Muribaculaceae uncultured bacterium* | str. zero | -0.1 | *Prevotellaceae NK3B31 group* | str. zero | 0 |
| *Alloprevotella uncultured bacterium* | str. zero | 0 | *Prevotellaceae NK3B31 group uncultured bacterium* | str. zero | -0.1 |
| *Catenibacterium uncultured bacterium* | str. zero | -0.3 | *Gastranaerophilales* | str. zero | -0.1 |
| *Enterococcus durans* | str. zero | 0.4 | *Asteroleplasma uncultured bacterium* | str. zero | 0 |
| *Lactobacillus mucosae* | str. zero | 0 | *Lactobacillus mucosae* | str. zero | 0 |
| *Phascolarctobacterium* | str. zero | -0.4 | *Lactobacillus salivarius* | str. zero | 0.2 |
| *Megasphaera* | str. zero | -0.3 | *Butyricicoccus pullicaecorum* | str. zero | 0 |
| *Megasphaera elsdenii* | str. zero | -0.1 | *Ruminococcus uncultured bacterium* | 68 | 1.4 |
| *Enterobacter* | str. zero | 0 | *Megasphaera uncultured bacterium* | str. zero | 0 |
| *Escherichia-Shigella uncultured bacterium* | str. zero | 0.2 | | | |
| *Morganella morganii* | str. zero | 0 | | | |
| *Treponema uncultured bacterium* | str. zero | 0 | | | |

**Table S2: Differentially abundant metabolic pathways reconstructed from 16S data by PICRUST2 between healthy controls and patients with inflammatory bowel diseases.** Differentially abundant pathways between healthy controls and IBD patients determined by ANCOM in descending order by the W-statistic ($W_{max}$ 390). Pathway entries from the MetaCyc database were reconstructed from 16S data. HC (healthy controls), IBD (inflammatory bowel disease), W (W-statistic), clr (centered log ratio).

| Pathway ID | W | clr | Up in | Description |
|---|---|---|---|---|
| THREOCAT-PWY | 382 | 1.15 | IBD | superpathway of L-threonine metabolism |
| PWY-5677 | 371 | -0.65 | HC | succinate fermentation to butanoate |
| PWY-6629 | 368 | 1.27 | IBD | L-tryptophan biosynthesis |
| P341-PWY | 367 | -0.67 | HC | glycolysis V (Pyrococcus) |
| PWY-7527 | 367 | -0.68 | HC | L-methionine salvage cycle III |
| PWY-4361 | 366 | -0.62 | HC | S-methyl-5-thio-_-D-ribose 1-phosphate degradation I |
| PWY-5005 | 365 | -0.66 | HC | biotin biosynthesis II |
| PWY-5088 | 365 | -0.76 | HC | L-glutamate degradation VIII (to propanoate) |
| ECASYN-PWY | 364 | 1.00 | IBD | enterobacterial common antigen biosynthesis |
| KDO-NAGLIPASYN-PWY | 363 | 0.95 | IBD | superpathway of (Kdo)2-lipid A biosynthesis |
| ARGDEG-PWY | 362 | 0.92 | IBD | superpathway of L-arginine, putrescine, and 4-aminobutanoate degradation |
| ORNARGDEG-PWY | 362 | 0.92 | IBD | superpathway of L-arginine and L-ornithine degradation |
| PWY-6142 | 355 | -0.63 | HC | gluconeogenesis II (Methanobacterium thermoautotrophicum) |
| ORNDEG-PWY | 354 | 0.83 | IBD | superpathway of ornithine degradation |
| METHGLYUT-PWY | 354 | 0.77 | IBD | superpathway of methylglyoxal degradation |
| HCAMHPDEG-PWY | 353 | 0.69 | IBD | 3-phenylpropanoate and 3-(3-hydroxyphenyl)propanoate degradation to 2-hydroxypentadienoate |
| PWY-6690 | 353 | 0.69 | IBD | cinnamate and 3-hydroxycinnamate degradation to 2-hydroxypentadienoate |
| KETOGLUCONMET-PWY | 351 | 0.79 | IBD | ketogluconate metabolism |
| PWY-7446 | 351 | 0.86 | IBD | sulfoquinovose degradation I |
| PWY-7254 | 350 | -0.63 | HC | TCA cycle VII (acetate-producers) |
| PWY-5910 | 349 | 0.89 | IBD | superpathway of geranylgeranyldiphosphate biosynthesis I (via mevalonate) |
| PWY-922 | 349 | 0.88 | IBD | mevalonate pathway I (eukaryotes and bacteria) |
| AST-PWY | 346 | 0.78 | IBD | L-arginine degradation II (AST pathway) |
| ENTBACSYN-PWY | 345 | 0.65 | IBD | enterobactin biosynthesis |
| ALL-CHORISMATE-PWY | 343 | 0.56 | IBD | superpathway of chorismate metabolism |
| PWY-5676 | 342 | -0.44 | HC | acetyl-CoA fermentation to butanoate II |
| PWY-6891 | 334 | -0.41 | HC | thiazole component of thiamine diphosphate biosynthesis II |
| PWY-2221 | 333 | -0.63 | HC | Entner-Doudoroff pathway III (semi-phosphorylative) |
| PWY-5177 | 324 | -0.47 | HC | glutaryl-CoA degradation |
